# Sex-based differences in the predictive significance of the waist circumference glucose index for future diabetes risk

**DOI:** 10.1038/s41598-025-07671-6

**Published:** 2025-07-01

**Authors:** Cheng Huang, Xiaoqing Yang, Junping Liu

**Affiliations:** 1 Department of Colorectal Surgery, First People’s Hospital of Xiaoshan District, Hangzhou, 311200 Zhejiang China; 2 Department of Endocrinology, First People’s Hospital of Xiaoshan District, Hangzhou, 311200 Zhejiang China

**Keywords:** Dynamic prediction, Fasting plasma glucose, Japanese population, Waist circumference, Endocrinology, Diabetes, Metabolic syndrome, Obesity

## Abstract

Diabetes is a chronic metabolic disorder that has become a growing global health concern. Waist circumference-glucose index (WyG) is an effective predictor of diabetes; however, its predictive performance in the Japanese population and potential sex-specific differences remain unexplored. This study evaluated the dynamic prediction capability of WyG for diabetes in Japanese individuals, focusing on the differences between sexes. We analyzed data from 15,464 Japanese adults initially without diabetes (7,034 women and 8,430 men). Multivariate Cox regression analysis was used to evaluate the association between WyG and diabetes. Sex-based subgroup analyses were conducted to assess the impact of potential confounders. Sensitivity analysis was conducted to exclude specific populations to test for robustness, and E-values were calculated to evaluate the impact of unmeasured cofounders. Predictive performance was assessed using time-dependent receiver operating characteristic curve analysis. WyG was significantly associated with diabetes in both sexes. This association remained consistent across female subgroups, whereas in males, it was influenced by age and the presence of fatty liver. The area under the curve values ranged from 0.73 to 0.78 in women and 0.73 to 0.77 in men. Sex-specific thresholds (WyG > 8.19 for women, > 8.32 for men) were identified, with greater relative risk elevation for individuals above the threshold. WyG is a reliable predictor of diabetes, with its predictive performance varying across sexes.

**Supplementary Information:**

The online version contains supplementary material available at 10.1038/s41598-025-07671-6.

## Introduction

Diabetes represents an increasing public health challenge that imposes considerable burdens on individuals and healthcare systems globally^1^. Factors such as aging, unhealthy lifestyles, socioeconomic disparities, and environmental influences are fueling the rise in diabetes, highlighting the urgent need for preventive measures^2^. In 2022, the age-standardized prevalence of diabetes worldwide was estimated at 13.9% for women and 14.3% for men^3^. However, some studies indicate that factors such as obesity, lack of physical activity, and unhealthy dietary patterns contribute to women having a higher risk of developing diabetes than men^4,5^. Women with diabetes often require higher insulin doses to maintain optimal glycemic control and are more susceptible to hypoglycemia^6^. However, although women have a more impaired postprandial glucose metabolism, men are more affected by fasting insulin resistance (IR)^7–9^.

Elevated fasting plasma glucose (FPG), obesity, and hypertension are among the leading risk factors contributing to reduced life expectancy^10^. High FPG and waist circumference (WC) are critical adverse factors leading to diabetes^11,12^. WC is a significant indicator of abdominal obesity, which is a defining characteristic of diabetes^13,14^. Therefore, WC is significantly associated with dysglycemia, including elevated FPG and glycated hemoglobin levels^15^. The triglyceride glucose-waist circumference (TyG-WC) index, calculated using FPG and WC, is a surrogate marker of IR and is significantly correlated with the incidence of diabetes^16^. In 2024, Zhao X et al.^17^ introduced a new index, the WC-glucose index (WyG) (ln [WC (cm) × FPG (mg/dL)/2]), derived from the TyG. They demonstrated that WyG exhibited greater predictive power for diabetes than both TyG and TyG-WC.

In addition to these risk factors, the prevalence and progression of diabetes also differ between sexes, and these differences change over time^18,19^. Men tend to engage in behaviors such as smoking and drinking, develop fatty liver, and experience obesity more frequently than women^20,21^. Additionally, hormonal fluctuations, which vary significantly according to sex, may further influence the risk over time^19^. Currently, the predictive efficacy of WyG in relation to sex or temporal variations remains unclear. Additionally, time-dependent receiver operating characteristic (ROC) curves can capture dynamic risk characteristics over time, which are crucial for chronic diseases, such as diabetes^22^.

However, the predictive performance of WyG in the Japanese population, particularly regarding sex-specific differences, remains unexplored. This study evaluated the dynamic prediction capability of WyG for diabetes in Japanese individuals without diabetes, focusing on the differences between sexes.

## Materials and methods

### Study design and population

This cohort study used information obtained from the NAGALA project, a comprehensive health assessment program initiated at Murakami Memorial Hospital, Japan, in 1994. The NAGALA project conducts extensive medical evaluations, with over 8,000 assessments performed annually. Furthermore, a large percentage of participants returned for follow-up examinations, making it possible to conduct a comprehensive longitudinal study of health data.

**Fig. 1:**
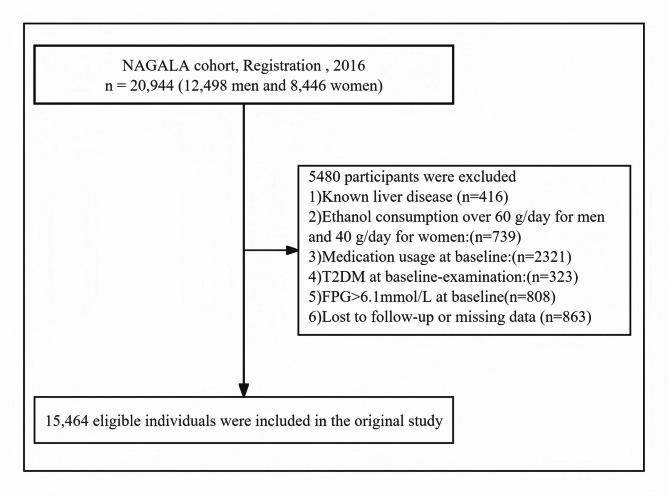
Flowchart of the selection process of study participants.

Individuals who participated in several health checkups between 2004 and 2015 were included in the dataset being processed for this research. Initially, 20,944 participants without diabetes were identified. Participants in the original study were excluded according to the following criteria: participants with pre-existing liver diseases, including viral hepatitis (*n* = 416), excessive alcohol consumption (*n* = 739), any medication usage (*n* = 2321), and incomplete data records (*n* = 863). Additionally, participants with impaired fasting glucose (*n* = 808) were excluded. Those with a diagnosis of diabetes (*n* = 323) were excluded as well. The final analysis included 15,464 participants (7,034 women and 8,430 men) after applying these criteria (Fig. 1).

### Data acquisition and collection

The data for this secondary study were sourced from the Dryad Digital Repository^23^ .Samples were processed by trained healthcare professionals using automated biochemical analyzers to ensure measurement accuracy. Measurements included demographic factors (age, sex), anthropometric parameters (body mass index [BMI], WC), systolic blood pressure (SBP), diastolic blood pressure (DBP), and comprehensive biochemical markers including gamma-glutamyl transferase (GGT), triglycerides (TG), FPG, total cholesterol (TC), alanine aminotransferase (ALT), high-density lipoprotein cholesterol (HDL-C), glycated hemoglobin (HbA1c), and aspartate aminotransferase (AST).

Comprehensive lifestyle information was collected using structured questionnaires and evaluated physical activity patterns as well as substance use. Participants who engaged in any type of sport more than once a week were defined as exercisers. Smoking status was classified into three groups: active smokers, past smokers, and those who have never smoked. Alcohol consumption was categorized into two groups based on weekly intake: non-drinkers and drinkers, with the drinkers group further classified into light, moderate, and heavy consumption groups^24^. Fatty liver disease was assessed by gastroenterology specialists using abdominal ultrasound examinations to identify key diagnostic criteria^25^. All data collection procedures adhered to standardized protocols to maintain consistency and integrity throughout the study. TyG-WC is an index of IR, composed of TG, FPG, and WC. WyG and TyG-WC were calculated using the following formulas^17,26^:

$$WyG=ln[WC(cm) \times FPG (mg/dL)/2]$$

$$TyG-WC=[TG (mg/dL) \times FPG (mg/dL)/2] \times WC (cm).$$

### Definition

Diabetes was identified based on any of the following criteria observed during follow-up: self-reported diabetes, FPG ≥ 7.0 mmol/L, or HbA1c ≥ 6.5%^27^.

### Statistical analysis

Continuous variables are summarized using means ± standard deviations or medians (interquartile ranges), whereas categorical variables are presented as frequencies (percentages). For continuous variables, analysis of variance or Kruskal–Wallis tests were conducted to compare groups, whereas categorical variables were analyzed using the chi-square test. Standardized differences were estimated using the inverse probability of treatment weighting approach, with values exceeding 10% deemed significant^28^. Multicollinearity was assessed using variance inflation factors (VIF), excluding variables with a VIF > 5 (Supplementary Table S1)^29^.

Univariate Cox regression was used to explore preliminary relationships, followed by multivariate Cox regression to examine diabetes risk per 0.1 standard deviation increase in WyG. Three sequential models were established: Model 1 was not adjusted for any variable; Model 2 was adjusted for demographics, BMI, fatty liver status, physical activity, smoking, and drinking; and Model 3 was further adjusted for biochemical markers and SBP. To evaluate the robustness of the primary outcomes, sensitivity analysis was conducted based on Model 3 across different study populations. To minimize potential lag effects and reverse causation, participants with a follow-up period of < 2 years were excluded from the first sensitivity analysis (Sensitivity Analysis 1). Subsequent analyses addressed specific confounding factors: Sensitivity Analysis 2 excluded individuals with hepatic steatosis, who were predisposed to diabetes progression, whereas Sensitivity Analysis 3 excluded participants with BMI ≥ 25 kg/m^2^. Finally, based on Model 3, E-value calculations were applied to estimate the lowest association strength of unmeasured confounders required to account entirely for the observed findings^30^. This analytical approach provides an estimate of the threshold at which residual confounders can negate the statistical significance of the identified associations.

Comprehensive subgroup analyses investigated interactions across demographic characteristics, Habit of exercise, presence of fatty liver, and lifestyle behaviors. Diabetes is a progressive condition. The predictive performance of TyG-WC, TyG, WC, and WyG were evaluated using time-dependent ROC curves over 2–12 year periods. This approach allowed for a dynamic and comprehensive evaluation of IR’s ability to predict diabetes. All analyses were conducted using EmpowerStats, with statistical significance set at *P* < 0.05.

## Results

### Baseline characteristics of the study

This study included 7,034 women and 8,430 men participants. Of them, 2,741 were diagnosed with fatty liver, and 2,524 had a BMI ≥ 25 kg/m^2^. The group that developed diabetes was older, had a larger WC, was predominantly male, and had a higher BMI. They also displayed worse metabolic and hepatic indicators, including elevated liver enzyme levels and significantly higher concentrations of TG, TC, and FPG. Participants with diabetes had lower levels of physical activity and a higher proportion of smoking and drinking. Collectively, these patterns indicate a clustering of metabolic risks and unhealthy lifestyle behaviors in individuals with diabetes (Table 1). The prevalence of diabetes increases with age in both males and females; however, the disparity between the two groups narrows, and after the age of 65, the prevalence rates are almost equivalent (Supplementary Table S2). Compared to women, men have more unhealthy factors, such as higher blood pressure, fatty liver, and higher proportions of smoking and drinking (Supplementary Table S3). Univariate regression analyses indicated that various risk factors were linked to the risk of diabetes in both sexes, although the strength of these associations differed according to sex (Supplementary Table S4).

**Table 1:** Baseline demographic, lifestyle, and laboratory characteristics of participants.

	Non-diabetic	Diabetic	Standardized difference, % (95% CI)
Participants (n)	15,091	373	
Male	8144 (53.97%)	286 (76.68%)	49 (39, 59)
Age, years	43.62 ± 8.89	47.14 ± 8.52	40 (30, 51)
BMI (kg/m^2^)	22.04 ± 3.07	25.03 ± 3.82	86 (76, 97)
WC, cm	76.26 ± 8.97	85.08 ± 10.20	92 (82, 102)
ALT (IU/L)	19.71 ± 14.05	31.36 ± 20.37	67 (56, 77)
AST (IU/L)	18.30 ± 8.58	22.45 ± 10.11	44 (34, 55)
GGT, IU/L	20.06 ± 17.83	30.57 ± 25.81	47 (37, 58)
HDL-C (mmol/L)	1.47 ± 0.40	1.19 ± 0.33	76 (66, 86)
TC (mmol/L)	5.12 ± 0.86	5.43 ± 0.90	35 (25, 46)
TG (mmol/L)	0.90 ± 0.64	1.50 ± 0.98	73 (62, 83)
HbA1c, %	5.16 ± 0.32	5.53 ± 0.37	107 (97, 118)
FPG (mg/dL)	92.77 ± 7.35	101.14 ± 6.43	121 (111, 132)
SBP (mmHg)	114.31 ± 14.91	122.03 ± 15.59	51 (40, 61)
DBP (mmHg)	71.44 ± 10.47	77.18 ± 10.23	55 (45, 66)
WyG	8.16 ± 0.17	8.36 ± 0.14	127 (116, 137)
Fatty liver	2518 (16.69%)	223 (59.79%)	99 (89, 109)
Habit of exercise	2658 (17.61%)	51 (13.67%)	11 (1, 21)
Alcohol consumption			21 (11, 31)
Non	11,539 (76.46%)	266 (71.31%)	
Light	1718 (11.38%)	40 (10.72%)	
Moderate	1323 (8.77%)	37 (9.92%)	
Heavy	511 (3.39%)	30 (8.04%)	
Smoking status			45 (35, 55)
None	8886 (58.88%)	145 (38.87%)	
Past	2875 (19.05%)	77 (20.64%)	
Current	3330 (22.07%)	151 (40.48%)	

Values are expressed as mean (standard deviation), median (quartile interval), or n (%). *ALT* alanine aminotransferase, *AST* aspartate aminotransferase, *BMI* body mass index, *DBP* diastolic blood pressure, *FPG* fasting plasma glucose, *GGT* gamma-glutamyl transferase, *HbA1c* hemoglobin A1c, *HDL-C* high-density lipoprotein cholesterol, *TC* total cholesterol, *TG* triglyceride, *SBP* systolic blood pressure, *WC* waist circumference, *WyG* waist-to-glucose index.

### Association between WyG and diabetes

To further assess the association between WyG and diabetes, a multivariate Cox regression analysis adjusted for multiple variables was performed (Table 2). Overall, the HR for Model 1 (unadjusted) was 2.31 (95% CI: 2.15–2.48), with comparable values for women (2.40, 95% CI: 2.13–2.70) and men (2.29, 95% CI: 2.10–2.51). The HRs increased after the demographic and lifestyle characteristics were taken into account in Model 2, with the overall HR being 2.45 (95% CI: 2.15–2.79); the HR for women was 2.54 (95% CI: 2.02–3.21), and the HR for men was 2.50 (95% CI: 2.13–2.93). Model 3 revealed decreased HRs: overall, 1.78 (95% CI: 1.55–2.03); women, 2.11 (95% CI: 1.65–2.70); and men, 1.72 (95% CI: 1.46–2.03). No statistically significant difference was observed when sex-based interaction testing was performed (*P* = 0.33). Based on Model 3 adjustments, the HRs per 0.1 SD increase in WyG were 1.78 overall, 2.11 in women, and 1.72 in men for diabetes incidence. The corresponding E values were 2.96, 3.64, and 2.83, respectively. These substantial E-values imply that it is improbable that any undetected confounding factors will significantly affect the established relationship between WyG levels and diabetes risk, thereby reinforcing the validity of these results.

**Table 2:** Multivariate Cox regression analyses in different models grouped by sex.

	HR (95%CI)			*P* for interaction	E-value
	Model 1	Model 2	Model 3		
Total	2.31 (2.15, 2.48)	2.45 (2.15, 2.79)	1.78 (1.55, 2.03)		2.96
Sex				0.33	
Female	2.40 (2.13, 2.70)	2.54 (2.02, 3.21)	2.11 (1.65, 2.70)		3.64
Male	2.29 (2.10, 2.51)	2.50 (2.13, 2.93)	1.72 (1.46, 2.03)		2.83

The models assessed the association between WyG (Per 0.1 SD increase) and diabetes.

Model 1: No covariates were adjusted. Model 2: Adjusted for age, BMI, drinking status, smoking status, fatty liver, exercise habits, and SBP. Model 3: Adjusted for Age, BMI, ALT, AST, exercise habits, GGT, HDL-C, TC, TG, HbA1c, fatty liver, drinking status, smoking status, and SBP.

**Fig. 2:**
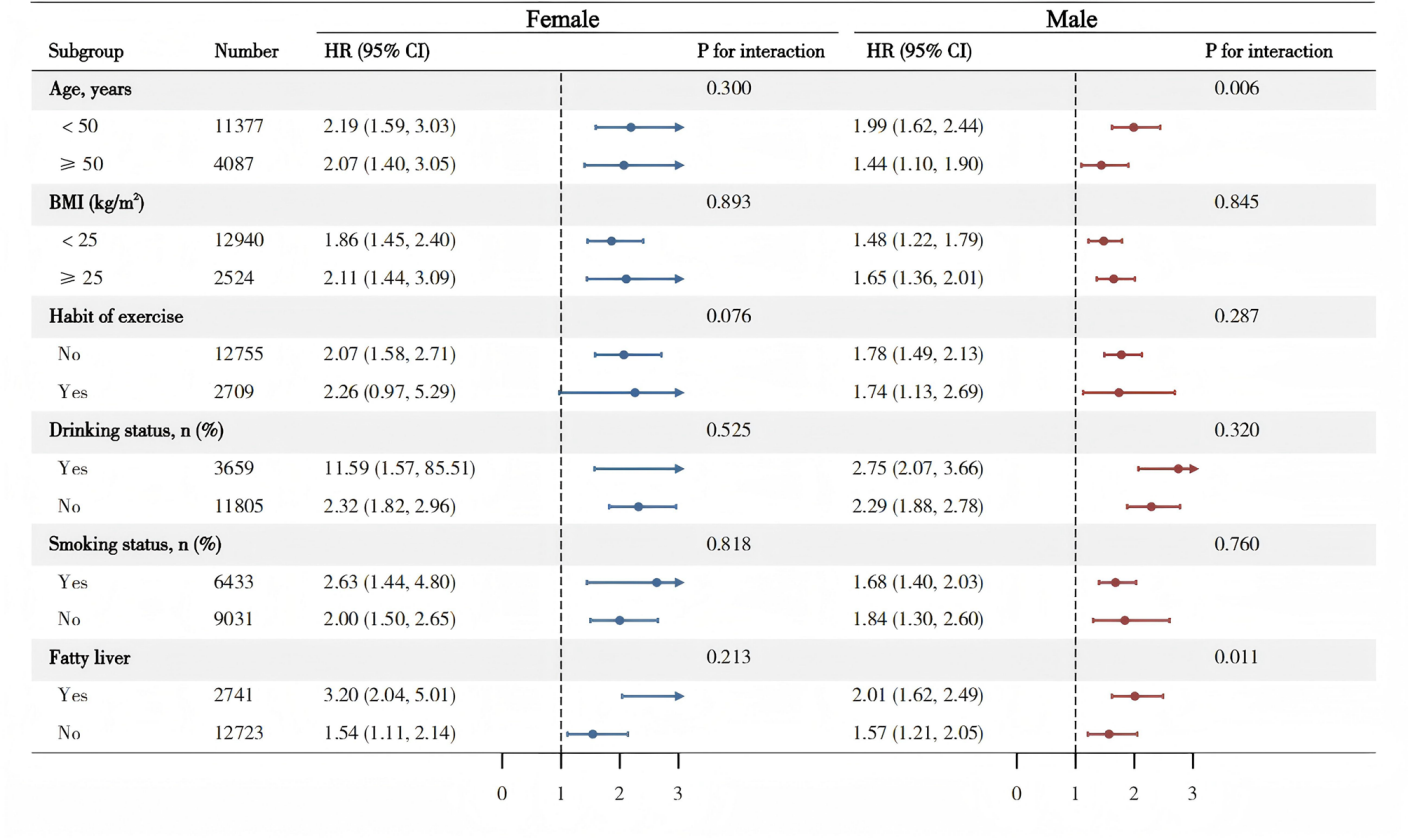
Subgroup analysis between WyG and diabetes. *WyG* waist-to-glucose index.

Subgroup analyses indicated a significant association between WyG and diabetes across both age categories (Fig. 2 and Supplementary Table S5). Notably, this relationship was more pronounced in individuals younger than 50 years of age. In female subgroups, the analyses demonstrated a consistent association between WyG and diabetes, with no statistically significant differences observed (P for interaction > 0.05). Conversely, analyses within male subgroups revealed significant interactions between age and the presence of fatty liver (P for interaction < 0.05), suggesting that these factors may modulate the relationship between WyG and diabetes in men. Specifically, younger males with fatty liver exhibited a stronger association with diabetes risk compared to their older counterparts and those without fatty liver. These findings highlight the critical importance of considering age and metabolic conditions, such as fatty liver disease, when assessing diabetes risk in male populations.

### Sensitivity analysis

Various approaches were applied to assess the reliability of the main results and the impact of specific groups (Supplementary Table S6). In Analysis 1, only individuals with a follow-up period exceeding 2 years were included to minimize the potential impact of reverse causation. Participants with fatty liver were excluded because of their predisposition to diabetes progression in Sensitivity Analysis 2. For Sensitivity Analysis 3, participants with a BMI below 25 kg/m^2^ were included to address the possible influence of adiposity as a confounding factor. The primary results remained consistently robust after a series of adjustments in each sensitivity analysis.

### Sex-specific predictive ability of WyG for diabetes

**Fig. 3:**
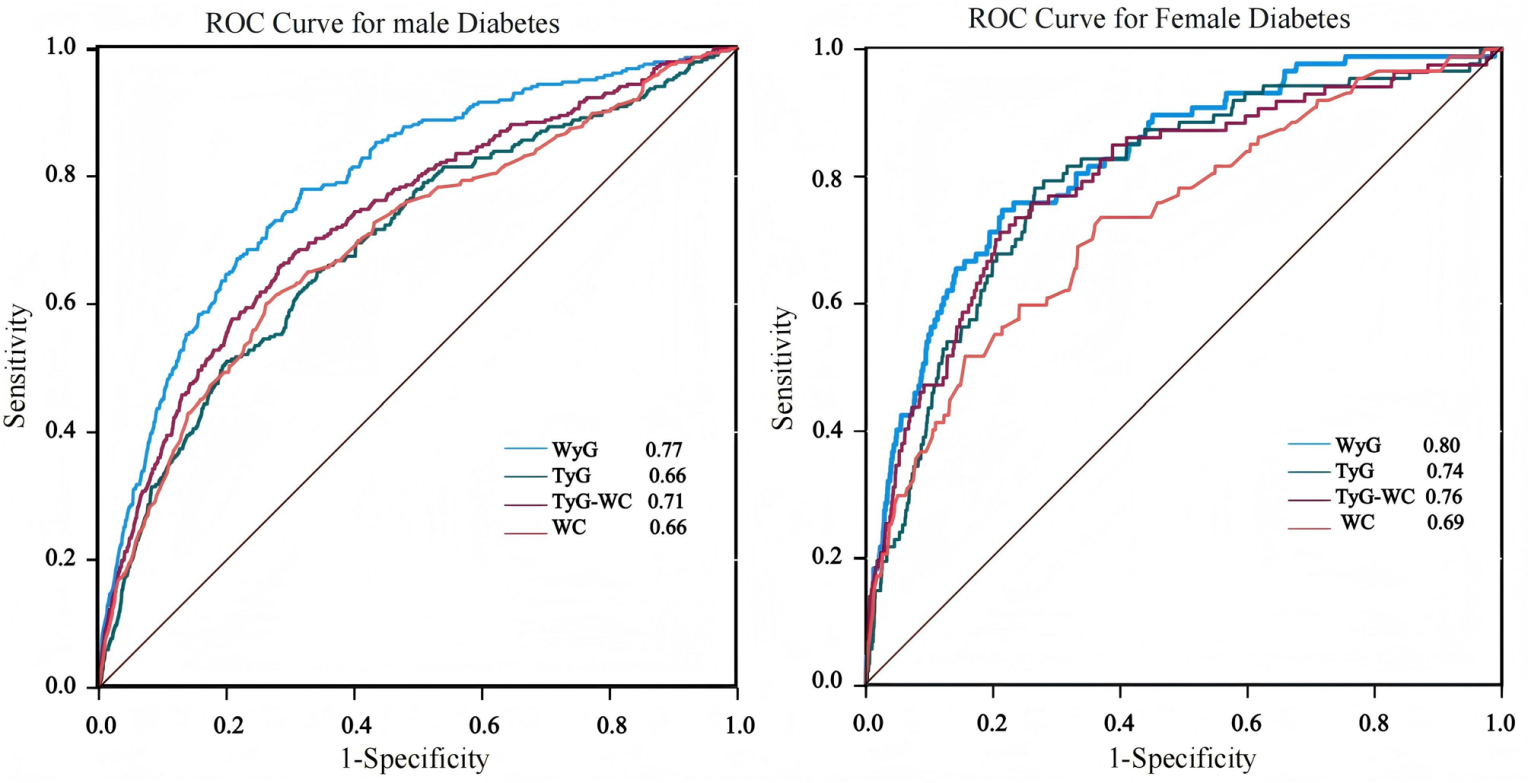
Compares the time-dependent ROC character of WyG, TyG, TyG-WC, and WC in predicting the onset of diabetes.

**Fig. 4:**
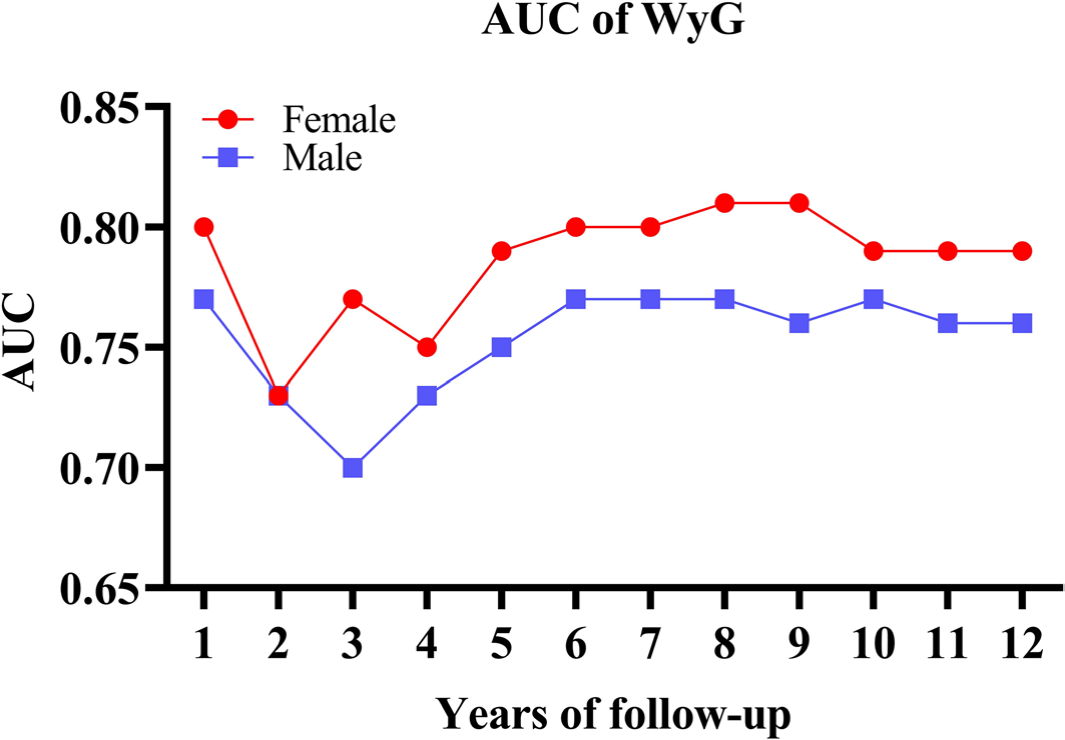
AUC fluctuations of the WyG for predicting the onset of diabetes in men and women. *AUC* area under the curve, *WyG* waist-to-glucose index.

**Fig. 5:**
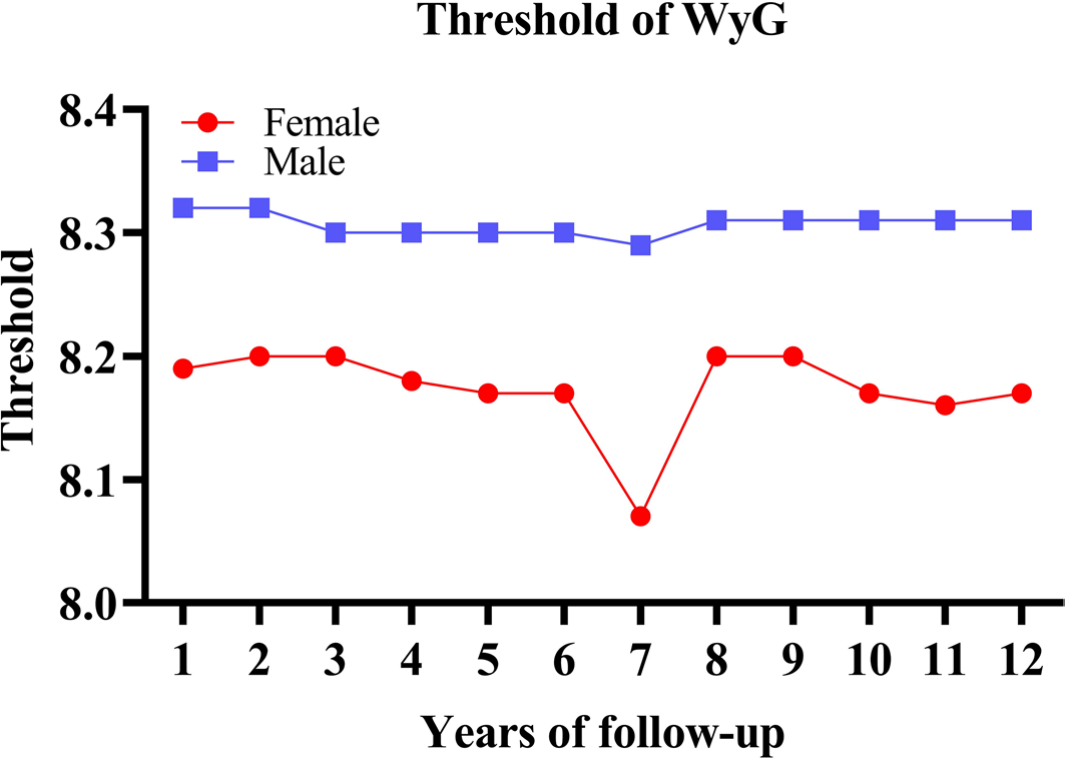
Threshold fluctuations of the WyG for predicting the onset of diabetes in men and women.

Among TyG, TyG-WC, and WC, WyG had the strongest ability to predict the onset of diabetes compared with the time-dependent ROC characteristics (Fig. 3 and Supplementary Table S7). Table 3 provides detailed metrics (thresholds, sensitivity, specificity, AUC) for diabetes prediction across 2–12 years. Figure 4 illustrates the temporal AUC trends, whiles Fig. 5 shows the threshold trends stratified by sex. During the follow-up period, the AUC values for women fluctuated between 0.73 and 0.81, whereas those for men ranged from 0.70 to 0.76. The total best thresholds for WyG were determined to be 8.19 for females and 8.32 for males. In a subsequent multivariable Cox regression analysis, adjusting for all variables (Model 3), females with a WyG greater than 8.19 exhibited an extraordinarily HR of 13.71 (95% CI: 8.59, 21.89). Similarly, males with a WyG exceeding 8.32 demonstrated a significant HR of 6.74 (95% CI: 5.18, 8.77). These findings indicate that both females and males with WyG values exceeding their best thresholds are at an elevated risk of developing diabetes, with females presenting a proportionately greater risk (Table 4).

**Table 3:** Time-dependent ROC curves for WyG predicting future diabetes risk for women and men.

	Women				Men			
	Best threshold	Sensitivity	Specificity	AUC	Best threshold	Sensitivity	Specificity	AUC
Total	8.19	0.63	0.81	0.80	8.32	0.72	0.70	0.77
2-years	8.20	0.55	0.82	0.73	8.32	0.65	0.69	0.73
3-years	8.20	0.64	0.82	0.77	8.30	0.68	0.64	0.70
4-years	8.18	0.62	0.78	0.75	8.30	0.71	0.64	0.73
5-years	8.17	0.72	0.76	0.79	8.30	0.75	0.64	0.75
6-years	8.17	0.72	0.76	0.80	8.30	0.77	0.65	0.77
7-years	8.07	0.91	0.54	0.80	8.29	0.79	0.64	0.77
8-years	8.20	0.66	0.81	0.81	8.31	0.73	0.70	0.77
9-years	8.20	0.65	0.82	0.81	8.31	0.72	0.70	0.76
10-years	8.17	0.69	0.77	0.79	8.31	0.73	0.70	0.77
11-years	8.16	0.69	0.75	0.79	8.31	0.72	0.69	0.76
12-years	8.17	0.70	0.77	0.79	8.31	0.71	0.70	0.76

*AUC* area under the curve, *ROC* receiver operating characteristic, *WyG* waist-to-glucose index.

**Table 4:** Multivariate Cox regression analyses in different models grouped by threshold.

	HR (95%CI)		
	Model 1	Model 2	Model 3
Total	2.31 (2.15, 2.48)	2.45 (2.15, 2.79)	1.78 (1.55, 2.03)
Sex			
Female	2.40 (2.13, 2.70)	2.54 (2.02, 3.21)	2.11 (1.65, 2.70)
≤ 8.19	Ref	Ref	Ref
> 8.19	13.71 (8.59, 21.89)	5.90 (3.25, 10.71)	3.74 (2.03, 6.90)
Male	2.29 (2.10, 2.51)	2.50 (2.13, 2.93)	1.72 (1.46, 2.03)
≤ 8.32	Ref	Ref	Ref
> 8.32	6.74 (5.18, 8.77)	3.58 (2.60, 4.92)	2.02 (1.46, 2.80)

The models assessed the association between WyG (Per 0.1 SD increase) and diabetes.

Model 1: No covariates were adjusted. Model 2: Adjusted for age, BMI, drinking status, smoking status, fatty liver, exercise habits, and SBP. Model 3: Adjusted for Age, BMI, ALT, AST, exercise habits, GGT, HDL-C, TC, TG, HbA1c, fatty liver, drinking status, smoking status, and SBP.

## Discussion

In this study, we systematically analyzed the ability of WyG to predict diabetes along with any sex-related differences. We found that the association between WyG and diabetes remained consistent even after performing multivariable Cox regression and sensitivity analyses. Additionally, the predictive ability of WyG for future diabetes development demonstrates differences between sexes.

The prevalence of diabetes is influenced by economic status, lifestyle, and genetic background^1^. From 1990 to 2022, the global diabetes prevalence significantly increased, rising from 6.8 to 14.3% in men and 6.9–13.9% in women^3^. East Asians face an elevated diabetes risk, with men being 2.84 times and women 2.18 times more likely to develop the condition^31^. WC is a simple yet effective indicator of abdominal obesity, a known risk factor for diabetes^15^. WC-related parameters have been used to predict diabetes, with TyG-WC being the most extensively studied^32^. TyG-WC, composed of three parameters (FPG, TG, and WC), is challenging to implement in large-scale screening. WC, as a surrogate marker of abdominal obesity, is closely associated with IR and abnormal fat distribution, whereas FPG directly reflects glycemic status in the fasting condition. Variations in an individual’s degree of obesity, adipose tissue distribution, hepatic function, and lifestyle factors may influence both WC and FPG, thereby further modifying the risk of developing diabetes. Therefore, WyG, a simplified parameter that combines only FPG and WC, may be more suitable for widespread applications. This index was initially developed by Zhao et al.^17^ and has demonstrated its potential for use in diabetes. WyG had a stronger predictive ability than WC, TyG, or TyG-WC. Aligning with previous findings, we found that the predictive ability of WyG for diabetes surpasses that of WC, TyG, and TyG-WC in both male and female populations. Notably, the AUC of TyG reached as high as 0.8 in the female population.

Despite the imbalance in the total number of diabetes events between men and women, with the incidence rate in men being approximately three times that in women, we still obtained similar adjusted hazard ratios results. While this outcome may initially seem contradictory, there are several biological and lifestyle-related factors that can help explain it. In this study population, men exhibit a higher burden of unhealthy factors, including an increased prevalence of fatty liver disease. Fatty liver disease can lead to impaired liver function, which in turn affects insulin sensitivity and utilization efficiency^33^. Fatty and inflammatory livers contribute to the heterogeneity in fatty liver-associated cardiometabolic risk by increasing glucose production, releasing procoagulant factors, causing dyslipidemia, and disrupting the regulation of liver-derived hormones and microRNAs^34,35^. Additionally, age and fatty liver disease are closely associated in patients with diabetes. Evidence suggests that these two factors may interact synergistically, thereby accelerating the progression of diabetes^36^. Notably, estrogen can reduce diacylglycerol accumulation in the liver, exert anti-inflammatory effects, and improve insulin sensitivity^37^. However, women may require higher doses or longer durations of glucose-lowering medications to achieve the desired therapeutic effects.

In addition to biological differences, lifestyle factors also play a significant role in our findings. Men exhibited higher alcohol consumption and lower physical activity levels, leading to increased prevalence of diabetes and fatty liver disease^20^. Both lifestyle and pharmaceutical therapies have been demonstrated to effectively mitigate the progression to diabetes^38,39^. Men showed greater improvements in indicators such as blood glucose and IR after lifestyle interventions; however, due to higher baseline fasting glucose levels and lower HDL cholesterol, the final incidence of diabetes was not significantly lower than that in women^40^. During the early diagnosis of diabetes, blood glucose levels and other biomarkers may change over time. Time-dependent ROC curves provide a more accurate assessment of the predictive ability of a model across different periods by constructing ROC curves at multiple time points^41^. In this study, during the follow-up period, the predictive ability of the WyG remained consistently stable in both men and women.

The pathophysiology that links obesity to diabetes involves multiple mechanisms. In individuals with obesity, adipose tissue inflammation triggers chronic systemic inflammatory responses, contributing to IR^42,43^. Adipose tissue generates reactive oxygen species, which induces metabolic disturbances, including obesity-related IR^44^. Mitochondrial dysfunction in adipose tissue further compounds these issues by impairing fatty acid oxidation, resulting in increased TG accumulation and worsened IR^45,46^. Additionally, mitochondrial-derived reactive oxygen species can damage cellular organelles, intensifying IR^47^. IR plays a crucial role in the development of numerous metabolic disorders, especially diabetes^48^.

With regard to the differences in the predictive abilities of WC, FPG, and composite indices derived from them such as WyG and TyG WC for diabetes, our study suggests that these disparities arise from multiple factors. Firstly, individual indices such as WC and FPG represent distinct aspects of metabolic risk, specifically body fat distribution and glucose metabolism, respectively. Their contributions to diabetes risk are rooted in fundamentally different pathophysiological mechanisms. For example, male participants in our study exhibited a higher prevalence of metabolic disturbances, including fatty liver and hypertension, which resulted in sex specific and baseline metabolic differences in risk assessment. Furthermore, composite indices like WyG, which integrate WC and FPG, are able to capture multidimensional metabolic disturbances. However, the predictive power of these indices also varies according to the interactions among variables and the structure of the formula used. Therefore, the observed differences among these indicators reflect not only their independent biological pathways related to diabetes risk, but also the impact of statistical methodology and the complexity of the formulas applied.

WyG is an easily obtainable parameter that makes it suitable for applications in public health initiatives and large-scale health screening programs. In clinical practice, the WyG cutoffs identified in this study could serve as valuable markers for diabetes risk stratification. For instance, in female patients, a WyG value exceeding 8.17–8.20 may indicate an elevated risk of developing diabetes, prompting clinicians to implement early interventions, such as lifestyle modifications involving dietary changes and increased physical activity. Similarly, in male patients, a WyG value above 8.29–8.31 could act as an early warning sign, guiding healthcare providers to take proactive measures to mitigate the risk. Our study also confirms that when WyG exceeds the best threshold, the risk of diabetes significantly increases in both men and women. Although there are sex differences in the ability to predict diabetes, the predictive performance of WyG for diabetes remains stable.

This study has several methodological strengths. First, the NAGALA cohort provided strong statistical power owing to its substantial population size and long-term follow-up. Second, the analytical approach utilized advanced statistical techniques, including sensitivity analyses and time-dependent ROC curves. Finally, from a sex perspective, this study investigated the differences in WyG’s ability to predict diabetes across different periods.

However, this study has some limitations. As this research was conducted in a particular location in Japan, the findings may not be generalizable to other ethnicities or regions. Moreover, this study lacks data on whether female participants are menopausal, which is an important factor related to the progression of diabetes. thereby impacting the precision of risk assessments. Furthermore, the reliance on ‘self-reported diabetes’ may introduce misclassification bias. This study may not have accounted for all potential confounding variables. Nevertheless, this limitation can be addressed by applying the E-value to assess the potential impact of unmeasured variables on the conclusions. Finally, given that the AUC is below 0.8, a significant limitation of this study is the reliance on a single predictor, which may oversimplify the multifactorial nature of diabetes risk and potentially overlook important interactions with other relevant variables.

## Conclusion

WyG serves as an effective tool for predicting future diabetes risk in non-diabetic Japanese populations, with variations in its predictive value observed between sexes. By collecting long-term data and analyzing WyG’s predictive ability across different age groups and lifestyle changes, its reliability and adaptability as a diabetes risk assessment tool can be further enhanced.

## Electronic supplementary material

Below is the link to the electronic supplementary material.

Supplementary Material 1

## Data Availability

The raw data can be downloaded from the DATADRYAD database (https://www.Datadryad.org). https://datadryad.org/stash/dataset/doi:10.5061%2Fdryad.8q0p192.
