# Supplementary material for: Sex-based differences in the predictive significance of the waist circumference glucose index for future diabetes risk
Source: Sci Rep. 2025 Jul 1;15:21477. doi: 10.1038/s41598-025-07671-6 (PMC12217243; doi:10.1038/s41598-025-07671-6)
Supplement: Supplementary file 1 — Supplementary Material 1 [file 41598_2025_7671_MOESM1_ESM.doc]

**Table S1. Collinearity screening between BRI and other variables.**

| Variance inflation factor | Variance inflation factor | | |
| --- | --- | --- | --- |
|  | Step 1 | Step 2 | Step 3 |
| Sex (M/F) | 2.1 | 2.1 | 2.1 |
| Age, years | 1.3 | 1.3 | 1.2 |
| Fatty liver | 1.6 | 1.6 | 1.6 |
| BMI (kg/m^2^) | 4.4 | 2.9 | 2.9 |
| ALT (IU/L) | 4.1 | 4.1 | 4.1 |
| AST (IU/L) | 3.3 | 3.3 | 3.3 |
| Habit of exercise | 1 | 1 | 1 |
| GGT (IU/L) | 1.5 | 1.5 | 1.5 |
| HDL-C (mmol/L) | 1.8 | 1.8 | 1.8 |
| TC (mmol/L) | 1.4 | 1.4 | 1.4 |
| TG (mmol/L) | 1.8 | 1.8 | 1.8 |
| HbA1c, % | 1.3 | 1.2 | 1.2 |
| smoking status | 1.3 | 1.3 | 1.3 |
| Drinking status | 1.4 | 1.4 | 1.4 |
| SBP (mmHg) | 5.6 | 5.6 | 1.4 |
| DBP (mmHg) | 5.7 | 5.7 | NA |

Note-1: Variance inflation factor = 1/(1-R^2^). Abbreviations as in Table 1.

Note-2: The variables with variance inflation factor >5 will be regarded as collinear variables and cannot be included in the multiple regression model.

**Table S2 Prevalence Rates of Diabetes According to Age and Gender**

| **Age, year** | **Female** | **Male** | **Total** |
| --- | --- | --- | --- |
| ≤35 | 0.50% | 1.41% | 1.00% |
| 35-40 | 0.68% | 2.58% | 1.70% |
| 40-45 | 1.47% | 4.00% | 2.82% |
| 45-50 | 1.36% | 4.81% | 3.11% |
| 50-55 | 2.18% | 4.91% | 3.69% |
| 55-60 | 1.84% | 3.32% | 2.73% |
| 60-65 | 2.48% | 5.30% | 4.42% |
| >65 | 5.36% | 5.71% | 5.59% |

**Table S3. Baseline demographic, lifestyle, and laboratory characteristics in participants classified by sex.**

|  | Female | Male | Standardized difference, % (95% CI) |
| --- | --- | --- | --- |
| Participants(n) | 7034 | 8430 |  |
| Age, years | 43.25 ± 8.76 | 44.09 ± 9.00 | 0.09 (0.06, 0.13) |
| BMI (kg/m2) | 21.01 ± 2.93 | 23.04 ± 2.98 | 0.69 (0.66, 0.72) |
| WC, cm | 71.68 ± 8.08 | 80.47 ± 7.91 | 1.10 (1.07, 1.13) |
| ALT (IU/L) | 15.05 ± 12.38 | 24.11 ± 14.57 | 0.67 (0.64, 0.70) |
| AST (IU/L) | 16.73 ± 8.99 | 19.79 ± 8.09 | 0.36 (0.33, 0.39) |
| GGT, IU/L | 13.65 ± 8.75 | 25.87 ± 21.72 | 0.74 (0.71, 0.77) |
| HDL-C (mmol/L) | 1.65 ± 0.38 | 1.30 ± 0.35 | 0.94 (0.91, 0.97) |
| TC (mmol/L) | 5.09 ± 0.88 | 5.16 ± 0.85 | 0.09 (0.05, 0.12) |
| TG (mmol/L) | 0.66 ± 0.41 | 1.12 ± 0.74 | 0.75 (0.72, 0.79) |
| HbA1c, % | 5.18 ± 0.32 | 5.16 ± 0.32 | 0.07 (0.03, 0.10) |
| FPG (mg/dl) | 89.81 ± 7.08 | 95.60 ± 6.66 | 0.84 (0.81, 0.88) |
| SBP (mmHg) | 109.36 ± 14.32 | 118.78 ± 14.14 | 0.66 (0.63, 0.69) |
| DBP (mmHg) | 67.64 ± 9.76 | 74.87 ± 9.96 | 0.73 (0.70, 0.77) |
| WyG | 8.07 ± 0.15 | 8.25 ± 0.13 | 1.27 (1.23, 1.30) |
| Fatty liver | 486 (6.91%) | 2255 (26.75%) | 0.55 (0.52, 0.58) |
| Habit of exercise | 1109 (15.77%) | 1600 (18.98%) | 0.08 (0.05, 0.12) |
| Alcohol consumption |  |  | 0.74 (0.71, 0.77) |
| Non/ | 6451 (91.71%) | 5354 (63.51%) |  |
| Light | 389 (5.53%) | 1369 (16.24%) |  |
| Moderate | 194 (2.76%) | 1166 (13.83%) |  |
| Heavy | 0 (0.00%) | 541 (6.42%) |  |
| Smoking status |  |  | 1.29 (1.26, 1.33) |
| None | 6139 (87.28%) | 2892 (34.31%) |  |
| Past | 441 (6.27%) | 2511 (29.79%) |  |
| Current | 454 (6.45%) | 3027 (35.91%) |  |

Values were expressed as mean (SD) or medians (quartile interval) or n (%). Abbreviations, BMI, body mass index; WC, waist circumference; ALT, alanine aminotransferase; AST, aspartate aminotransferase; GGT, gamma-glutamyl transferase; HDL-C, high-density lipoprotein cholesterol; TC, total cholesterol; TG, triglyceride; HbA1c, hemoglobin A1c; FPG, fasting plasma glucose; SBP, systolic blood pressure; DBP, diastolic blood pressure; WyG, lipid accumulation product.

**Table S4. Univariate regression analysis of the associations between WyG and baseline variables in different Gender**

|  | **HR (95%CI)** | |
| --- | --- | --- |
| Gender | **Female** | **Male** |
| Age, years | 1.08 (1.05, 1.11) | 1.05 (1.03, 1.06) |
| Fatty liver | 14.53 (9.53, 22.17) | 4.88 (3.83, 6.21) |
| BMI (kg/m2) | 1.31 (1.25, 1.37) | 1.21 (1.18, 1.24) |
| WC, cm | 1.11 (1.10, 1.13) | 1.08 (1.07, 1.10) |
| ALT (IU/L) | 1.00 (1.00, 1.01) | 1.02 (1.02, 1.03) |
| AST (IU/L) | 1.01 (1.00, 1.01) | 1.03 (1.03, 1.04) |
| Habit of exercise | 0.83 (0.45, 1.52) | 0.71 (0.51, 1.00) |
| GGT, IU/L | 1.03 (1.02, 1.03) | 1.01 (1.01, 1.01) |
| HDL-C (mmol/L) | 0.14 (0.07, 0.28) | 0.20 (0.13, 0.30) |
| TC (mmol/L) | 1.81 (1.48, 2.23) | 1.36 (1.20, 1.55) |
| TG (mmol/L) | 2.95 (2.44, 3.56) | 1.62 (1.49, 1.76) |
| HbA1c, % | 79.44 (40.48, 155.92) | 53.28 (37.08, 76.55) |
| Alcohol consumption | 0.69 (0.28, 1.71) | 0.89 (0.70, 1.14) |
| Smoking status |  |  |
| Never | Ref | Ref |
| Past | 1.35 (0.55, 3.36) | 1.11 (0.80, 1.53) |
| Curren | 2.93 (1.59, 5.42) | 1.69 (1.27, 2.23) |
| FPG (mg/dl) | 1.20 (1.16, 1.23) | 1.20 (1.17, 1.22) |
| SBP (mmHg) | 1.03 (1.02, 1.04) | 1.03 (1.02, 1.03) |
| DBP (mmHg) | 1.05 (1.03, 1.07) | 1.04 (1.03, 1.05) |
| WyG（Per 0.1 SD increase） | 2.40 (2.13, 2.70) | 2.29 (2.10, 2.51) |
|

Abbreviations, BMI, body mass index; WC, waist circumference; ALT, alanine aminotransferase; AST, aspartate aminotransferase; GGT, gamma-glutamyl transferase; HDL-C, high-density lipoprotein cholesterol; TC, total cholesterol; TG, triglyceride; HbA1c, hemoglobin A1c; FPG, fasting plasma glucose; SBP, systolic blood pressure; DBP, diastolic blood pressure; WyG, waist to glucose index.

**Table S5. Gender Composition Based on Demographic and Lifestyle Factors**

| **Sub-group** | **Sex= Female** | **Sex= Male** | **Total** |
| --- | --- | --- | --- |
| Age, years <50 | 5307 | 6070 | 11377 |
| Age, years ≥50 | 1727 | 2360 | 4087 |
| **BMI (kg/m²) <25** | 6406 | 6534 | 12940 |
| **BMI (kg/m²) ≥25** | 628 | 1896 | 2524 |
| **Habit of exercise = No** | 5925 | 6830 | 12755 |
| **Habit of exercise = Yes** | 1109 | 1600 | 2709 |
| **Alcohol consumption = No** | 6451 | 5354 | 11805 |
| **Alcohol consumption = Yes** | 583 | 3076 | 3659 |
| **Smoking status = No** | 6139 | 2892 | 9031 |
| **Smoking status = Yes** | 895 | 5538 | 6433 |
| **Fatty liver = No** | 6548 | 6175 | 12723 |
| **Fatty liver = Yes** | 486 | 2255 | 2741 |

**Table S6. Sensitivity analyses: diabetes risk associated with baseline TyG parameters in different test populations**

|  |  | |  | HR (95%CI) | |  |  |
| --- | --- | --- | --- | --- | --- | --- | --- |
|  | Sensitivity Analysis 1 |  |  | Sensitivity Analysis 2 |  |  | Sensitivity Analysis 3 |
| Total | 1.78 (1.54, 2.06) |  |  | 1.60 (1.38, 1.86) |  |  | 1.51 (1.23, 1.85) |
| Sex |  |  |  |  |  |  |  |
| Female | 2.11 (1.62, 2.76) |  |  | 1.86 (1.45, 2.40) |  |  | 1.57 (1.13, 2.18) |
| Male | 1.71 (1.43, 2.05) |  |  | 1.48 (1.22, 1.79) |  |  | 1.56 (1.19, 2.04) |

HR, hazard ratio; CI, confidence interval. Other abbreviations are shown in Table 1.

(1) Sensitivity-1: Participants with a follow-up period <2 years were excluded (n=12,545).

(2) Sensitivity-2: Participants with BMI ≥ 25 kg/m2 at baseline were excluded (n=12,940).

(3) Sensitivity-3: Participants diagnosed with the fatty liver at baseline were excluded (n=12,723).

Sensitivity Analysis 1 was adjusted for age, BMI, ALT, AST, exercise habits, GGT, HDL-C, TC, TG, HbA1c, fatty liver, drinking status, smoking status, and SBP.

Sensitivity Analysis 2 was adjusted for sex, age, TC, HDL-C, HbA1c, SBP, fatty liver, exercise habits, drinking status, and smoking status.

Sensitivity Analysis 3 was adjusted for sex, age, BMI, TC, HDL-C, HbA1c levels, SBP, exercise habits, drinking status, and smoking status.

**Table S7. Time-dependent ROC curves for TyG-WC, TyG, and WC in predicting future diabetes risk for women and men**

|  | Women | | | |  | Men | | | |
| --- | --- | --- | --- | --- | --- | --- | --- | --- | --- |
|  | Best threshold | Sensitivity | Specificity | AUC |  | Best threshold | Sensitivity | Specificity | AUC |
| **TyG-WC** |  |  |  |  |  |  |  |  |  |
| 2-years | 616.46 | 0.62 | 0.79 | 0.71 |  | 737.51 | 0.49 | 0.77 | 0.66 |
| 4-years | 603.96 | 0.60 | 0.75 | 0.69 |  | 732.14 | 0.53 | 0.76 | 0.66 |
| 6-years | 559.34 | 0.83 | 0.59 | 0.76 |  | 730.77 | 0.57 | 0.75 | 0.70 |
| 8-years | 559.34 | 0.84 | 0.59 | 0.78 |  | 716.33 | 0.63 | 0.72 | 0.72 |
| 10-years | 605.73 | 0.69 | 0.76 | 0.77 |  | 704.76 | 0.65 | 0.68 | 0.70 |
| 12-years | 600.99 | 0.74 | 0.76 | 0.79 |  | 710.14 | 0.59 | 0.70 | 0.68 |
| **TyG** |  |  |  |  |  |  |  |  |  |
| 2-years | 7.90 | 0.77 | 0.64 | 0.65 |  | 8.50 | 0.64 | 0.65 | 0.64 |
| 4-years | 7.98 | 0.71 | 0.69 | 0.69 |  | 8.51 | 0.61 | 0.66 | 0.64 |
| 6-years | 7.98 | 0.73 | 0.69 | 0.73 |  | 8.43 | 0.64 | 0.61 | 0.66 |
| 8-years | 7.98 | 0.73 | 0.69 | 0.74 |  | 8.49 | 0.63 | 0.65 | 0.67 |
| 10-years | 8.00 | 0.74 | 0.71 | 0.74 |  | 8.24 | 0.761 | 0.49 | 0.66 |
| 12-years | 8.01 | 0.78 | 0.72 | 0.77 |  | 8.50 | 0.57 | 0.67 | 0.64 |
| **WC** |  |  |  |  |  |  |  |  |  |
| 2-years | 76.20 | 0.54 | 0.75 | 0.64 |  | 81.00 | 0.63 | 0.56 | 0.61 |
| 4-years | 76.50 | 0.46 | 0.76 | 0.62 |  | 84.00 | 0.54 | 0.71 | 0.62 |
| 6-years | 78.00 | 0.49 | 0.81 | 0.69 |  | 84.00 | 0.56 | 0.71 | 0.65 |
| 8-years | 74.20 | 0.66 | 0.67 | 0.72 |  | 84.00 | 0.55 | 0.71 | 0.66 |
| 10-years | 73.90 | 0.67 | 0.65 | 0.70 |  | 84.00 | 0.55 | 0.72 | 0.66 |
| 12-years | 73.20 | 0.75 | 0.64 | 0.73 |  | 83.90 | 0.56 | 0.70 | 0.65 |

AUC, area under the curve; ROC, receiver operating characteristic; WyG, waist-to-glucose index
